# Comprehensive Analysis of *CRIP1* Expression in Acute Myeloid Leukemia

**DOI:** 10.3389/fgene.2022.923568

**Published:** 2022-07-22

**Authors:** Yan Gao, Jin-Yuan Li, Jia-Ying Mao, Jia-Fan Zhou, Lu Jiang, Xue-Ping Li

**Affiliations:** ^1^ Department of Medical Oncology, Sun Yat-sen University Cancer Center, Guangzhou, China; ^2^ State Key Laboratory of Oncology in South China, Collaborative Innovation Center for Cancer Medicine, Guangzhou, China; ^3^ Department of Hematologic Oncology, Sun Yat-sen University Cancer Center, Guangzhou, China; ^4^ Department of Nephrology, The Sixth Affiliated Hospital, Sun Yat-sen University, Guangzhou, China; ^5^ Shanghai Institute of Hematology, State Key Laboratory of Medical Genomics, National Research Center for Translational Medicine at Shanghai, Ruijin Hospital, Shanghai Jiao Tong University School of Medicine, Shanghai, China

**Keywords:** AML, immune infiltration, *CRIP1*, gene expression profiling, single-cell RNA sequencing

## Abstract

Acute myeloid leukemia (AML) is a highly heterogeneous hematological malignancy that imposes great challenges in terms of drug resistance and relapse. Previous studies revealed heterogeneous leukemia cells and their relevant gene markers, such as *CRIP1* as clinically prognostic in t (8;21) AML patients. However, the expression and role of *CRIP1* in AML are poorly understood. We used the single-cell RNA sequencing and gene expression data from t (8;21) AML patients to analyze the immune and regulation networks of *CRIP1*. Two independent cohorts from GSE37642 and The Cancer Genome Atlas (TCGA) datasets were employed as validation cohorts. In addition, the methylation data from TCGA were used to analyze the methylation effect of the *CRIP1* expression. Gene expression profile from t (8;21) AML patients showed that the *CRIP1*-high group exhibited an enrichment of immune-related pathways, including tumor necrosis factor (TNF)α signaling via nuclear factor kappa B (NFκB) pathways. Further studies using CIBERSORT showed that the *CRIP1*-high group had a significantly higher infiltration of exhausted CD8 T cells and activated mast cells. The *CRIP1* expression was validated in the GSE37642-GPL96, GSE37642-GPL570, and TCGA datasets. In addition, with the methylation data, four CpG probes of *CRIP1* (cg07065217, cg04411625, cg25682097, and 11763800) were identified as negatively associated with the *CRIP1* gene expression in AML patients. Our data provide a comprehensive overview of the regulation of *CRIP1* expression in AML patients. The evaluation of the TNFα-NFκB signaling pathway as well as the immune heterogeneity might provide new insights for exploring improvements in AML treatment.

## Introduction

Acute myeloid leukemia (AML) is a highly heterogeneous hematological malignancy characterized by the malignant proliferation of clonal myeloid precursor cells that progress rapidly (Döhner et al., 2015). t (8;21) AML related to the *RUNX1*-*RUNX1T1* (*AML1-ETO*, AE) fusion gene, accounts for approximately 7% of adult primary AML (Arber et al., 2016; Faber et al., 2016; Papaemmanuil et al., 2016; Döhner et al., 2017). At present, with standard chemotherapy (anthracycline combined with cytarabine) , t (8;21) AML patients under the age of 60 could achieve a complete remission rate (CR) of more than 80%. However, over 40% of t (8;21) AML patients relapsed and responded poorly to salvage chemotherapy, with the 5-year overall survival (OS) rate less than 50% (Zhu et al., 2013; Hospital et al., 2014; Papaemmanuil et al., 2016).

AML leukemia cells can evade the surveillance of the bone marrow (BM) immune system. Immune dysfunction of AML, including the downregulation of major histocompatibility complex class II genes, is associated with relapse (Christopher et al., 2018). In addition, the impaired immune cells (such as T cells and NK cells) of the AML BM microenvironment are prognostic of clinical outcome (Fauriat et al., 2007; Lion et al., 2012; van Galen et al., 2019; Tang et al., 2020).

In recent years, with the rapid development of single-cell sequencing, researchers have been able to discover previously unknown cells, especially the heterogeneity of immune-infiltrating cells in the tumor microenvironment (Guo et al., 2018; Li et al., 2019; Zhang et al., 2020). Through single-cell RNA-seq (scRNA-seq) analysis, we previously identified the CD34^+^CD117^dim^ leukemia cells and their characteristic gene markers (*LGALS1*, *EMP3*,and *CRIP1*) in t (8;21) AML patients (Jiang et al., 2020). A retrospective analysis confirmed that the proportion of the CD34^+^CD117^dim^ cells was clinically relevant to the OS.

Among the markers of the CD34^+^CD117^dim^ cells, cysteine-rich intestinal protein (*CRIP1*) belongs to the LIM/double-zinc finger protein family and is abnormally expressed in a variety of tumors, including breast cancer, colorectal tumors, and thyroid cancer (Ludyga et al., 2013; Li et al., 2017; He et al., 2019; Zhang et al., 2019). *CRIP1* may have tumor-type-specific oncogenic or tumor-suppressive properties (Sun et al., 2021). In detail, using the RNA-seq data from the Cancer Genome Atlas (TCGA) AML project, we discovered that *CRIP1* was highly expressed in AML patients, including the M0–M7 subtypes (Li et al., 2021). Using the COX regression model, our previous research demonstrated *CRIP1* as an independent risk factor for the OS of t (8;21) AML patients (Li et al., 2021). However, the regulation of *CRIP1* expression in t (8;21) AML leading to poor outcomes as well as the clinical significance of *CRIP1* in AML remain unclear. This study analyzed the immune infiltration state as well as the epigenetic effect on the *CRIP1* expression in AML.

## Methods

### Data Collection

scRNA-seq data via 10x Genomics and RNA-seq data of t (8;21) AML patients were downloaded from our previous studies, which were deposited in the National Omics Data Encyclopedia (http://www.biosino.org/node/project/detail/OEP000629). Detailed treatment information was provided, as previously described (Jiang et al., 2020). This study was approved by the Ruijin Hospital Review Board, and informed consent was obtained from all patients in accordance with the Declaration of Helsinki. Clinical and transcriptome information of TCGA LAML was downloaded from the online database (https://portal.gdc.cancer.gov/) (Cancer Genome Atlas Research et al., 2013). GSE37642 (AMLCG 1999) and GSE116438 could be downloaded from the Gene Expression Omnibus databases.

### Gene Expression Analysis

To quantify the *RUNX1-RUNX1T1* transcript, Salmon (Patro et al., 2017) were used to determine the transcripts per kilobase million value, as previously described (Jiang et al., 2020). Gene set enrichment analysis (GSEA) was performed using the GSEA software (www.broadinstitute.org/gsea) and the Molecular Signatures Database, as described previously (Jiang et al., 2020; Li et al., 2021). The ingenuity pathway analysis (IPA) software (Qiagen Redwood City) with the default parameters was employed to identify the upstream regulator.

### Protein–Protein Interaction Network Construction

The signature of CD34^+^CD117^dim^ populations was extracted from previous scRNA-seq data with the criteria of an average log Fold Change (avg_logFC) > 0.5 and an adjusted *p* value <0.05. The Search Tool for the Retrieval of Interacting Genes/Proteins (STRING) database (https://cn.string-db.org) was then used to predict the protein–protein interaction (PPI) network (Szklarczyk et al., 2021) of the signature of CD34^+^CD117^dim^ populations. The minimum required interaction score was 0.4, and the interaction predictions included text mining, experiments, databases, etc.

### Immune Infiltration Analysis in Acute Myeloid Leukemia Dataset

To study the enrichment of immune cells in the BM microenvironment of AML patients, we used CIBERSORT (Newman et al., 2015). For each sample, relative abundance of 22 types of infiltrating immune cells, including T, B, and NK cells, and macrophages were analyzed. A correlation between immune cells inferred by CIBERSORT and *CRIP1* expression was evaluated using Spearman’s correlation. The distribution of immune cells between high- and low-*CRIP1* groups was compared using two-sided Wilcoxon test.

### Cancer Cell Line Encyclopedia Database

The gene expression data of cancer cell line encyclopedia (CCLE) were extracted from the dataset (https://portals.broadinstitute.org/ccle/data) (Barretina et al., 2012) for analysis.

### MEXPRESS, cBioPortal, and Cistrome

MEXPRESS (http://mexpress.be) (Koch et al., 2019) and cBioPortal (http://cbioportal.org/) (Gao et al., 2013) were utilized to explore the association between the *CRIP1* expression and methylation levels at multiple DNA sites. The CistromeDB Toolkit (http://dbtoolkit.cistrome.org/) (Zheng et al., 2019) was used to analyze a large database of uniformly analyzed published ChIP-seq data.

### Genomics of Drug Sensitivity in Cancer Database

RNA-seq and predicted chemotherapeutic response were downloaded from the Genomics of Drug Sensitivity in Cancer (GDSC) database (https://www.cancerrxgene.org/) (Yang et al., 2013). The prediction process was implemented by R package “pRRophetic.” The samples’ half-maximal inhibitory concentration (IC50) was estimated using ridge regression. All the parameters were set as the default values. Using the batch effect of the combat and tissue type of all tissue, the duplicate gene expression was summarized as the mean value.

### Statistical Analysis

Univariate COX regression analysis and forest analysis were performed using the forestplot package. The Kaplan–Meier method was employed to estimate the probabilities of OS and relapse-free survival, and the log-rank test was used to compare the *p* value. Statistical analyses were performed using R software (version 4.0.2, https://www.r-project.org/).

## Results

### Clinical Features and Outcomes for *CRIP1* in Acute Myeloid Leukemia Patients

The previous scRNA-seq data of nine t (8;21) AML patients were used (Jiang et al., 2020). The signatures of CD34^+^CD117^dim^ populations were extracted using the criteria of an average log Fold Change (avg_logFC) >0.5 and an adjusted *p* value <0.05 (Supplementary Table S1). We then used the STRING database (https://cn.string-db.org) to analyze the PPI network (Supplementary Figure S1).

Out of all hub genes, *CRIP1* was demonstrated to be the prognostic of t (8;21) AML patients using the COX regression model, and it was highly expressed in AML subtypes, including M0–M7 (Li et al., 2021). For AML subtypes M0 and M2, patients with a higher *CRIP1* expression showed a significantly worse OS (Supplementary Figure S2). In addition, we analyzed the correlation of *CRIP1* expression with clinical characteristics, including age, sex, white blood cell (WBC) count, and gene mutations. The AML patients with a higher WBC count had a significantly higher *CRIP1* expression (*p* < 0.001), while the gene mutations, including *TP53*, *RUNX1,* and *FLT3-ITD*, had no significant correlation with *CRIP1* expression (Supplementary Figure S3). Here, based on previous research, we attempted to reveal the expression and regulation of *CRIP1* in AML patients.

### Immune Dysregulation in the *CRIP1*-High Group of t (8;21) AML Patients

**FIGURE 1:**
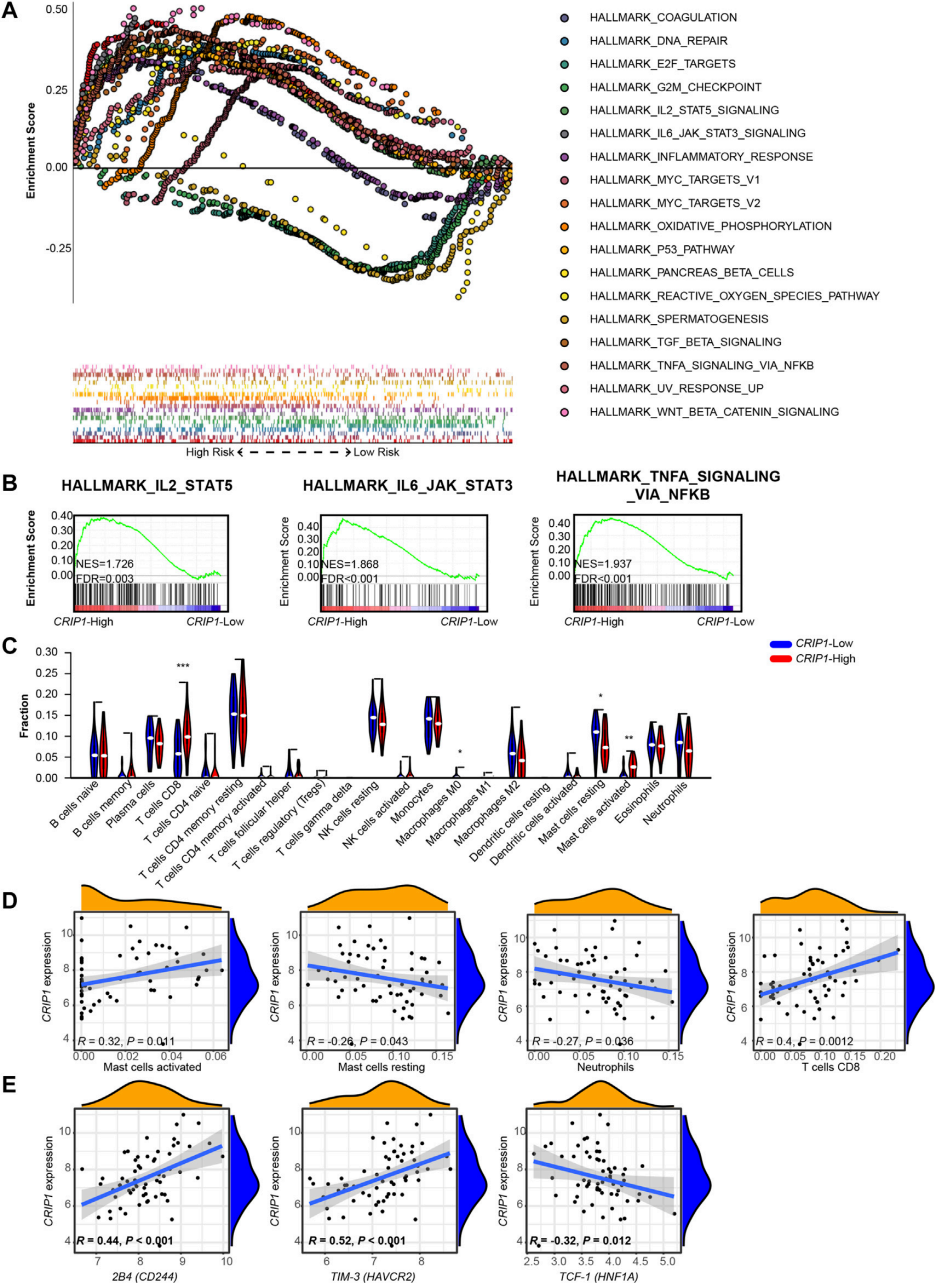
Gene set enrichment analysis (GSEA) and immune infiltration analysis of *CRIP1* expression in the t (8;21) acute myeloid leukemia (AML) patients. **(A)** Top enriched pathways in the high- (*n* = 31) and low- (*n* = 31) *CRIP1* expression groups from the 62 *de novo* t (8;21) AML patients. They were classified based on the median level of *CRIP1* expression. **(B)** Representative GSEA plots showing the activated immune-related pathways in the high-*CRIP1* expression group from the t (8;21) AML patients. Normalized enrichment score (NES) and false discovery rate (FDR) values are given. **(C)** Proportion of immune infiltrated cells between high- and low- *CRIP1* expression of t (8;21) AML patients based on the CIBERSORT algorithm. *, *p* < 0.05; **, *p* < 0.01; and ***, *p* < 0.001. Statistical significance was determined using two-sided Wilcoxon test. **(D)** Correlation analysis of *CRIP1* expression with the proportion of differential immune cells of t (8;21) AML patients. Spearman’s correlation analysis and correlation coefficient (*R*) are shown. **(E)** Correlation analysis of *CRIP1* expression with the exhaustion marker of CD8 T cells. Spearman’s correlation analysis and correlation coefficient (*R*) are shown.

First, to explore the upregulation pathway of *CRIP1* in t (8;21) AML patients, the gene expression profile from previous RNA-seq data of 62 *de novo* t (8;21) AML patients was analyzed (Jiang et al., 2020). The patients were classified into high- and low-*CRIP1* groups according to the median value of *CRIP1* expression. GSEA results revealed that the *CRIP1*-high group (*n* = 31) had an enrichment of immune-related pathway, such as IL2-STAT5 signaling, IL6-JAK-STAT3 signaling, and tumor necrosis factor (TNF)α signaling via the nuclear factor kappa B (NFκB) pathways (Figures 1A,B). For the *CRIP1*-low group (*n* = 31), we observed a significant enrichment of DNA repair and E2F target pathway (Figure 1A). This highly activated immune-related pathway indicated immune dysregulation in the *CRIP1*-high group of t (8;21) AML patients.

Thus, the immune infiltration of the 22 immune cell proportions in t (8;21) AML patients was analyzed and compared based on the CIBERSORT algorithm (Newman et al., 2015). Of the 22 immune cells, the *CRIP1*-high group had a significantly higher infiltration of T cells CD8 and Mast cells activated (Figure 1C), whereas the *CRIP1*-low group had a significantly higher proportion of macrophages M0 and mast cells resting (Figure 1C). Furthermore, Spearman’s correlation analysis demonstrated that the *CRIP1* expression had a positive correlation with the mast cells activated (*R* = 0.32, *p* = 0.011) and CD8 T cells (*R* = 0.4, *p* = 0.0012). In contrast, the *CRIP1* expression had a negative correlation with resting mast cells (*R* = −0.26, *p* = 0.043) and neutrophils (*R* = −0.27, *p* = 0.036) (Figure 1D). The higher proportion of CD8 T cells and inferior outcome of the *CRIP1*-high group made us further explore the functional status of T cells. The expression of the exhaustion T cell markers, including 2B4, TIM-3, and TCF-1, was analyzed. The *CRIP1* expression had a positive correlation with 2B4 (*R* = 0.44, *p* < 0.001) and TIM-3 (*R* = 0.52, *p* < 0.001) and a negative correlation with TCF-1 (HNF1A) (*R* = −0.32, *p* = 0.012) (Figure 1E).

### *CRIP1* Expression Could be Regulated by the TNFα–NFκB Pathway

**FIGURE 2:**
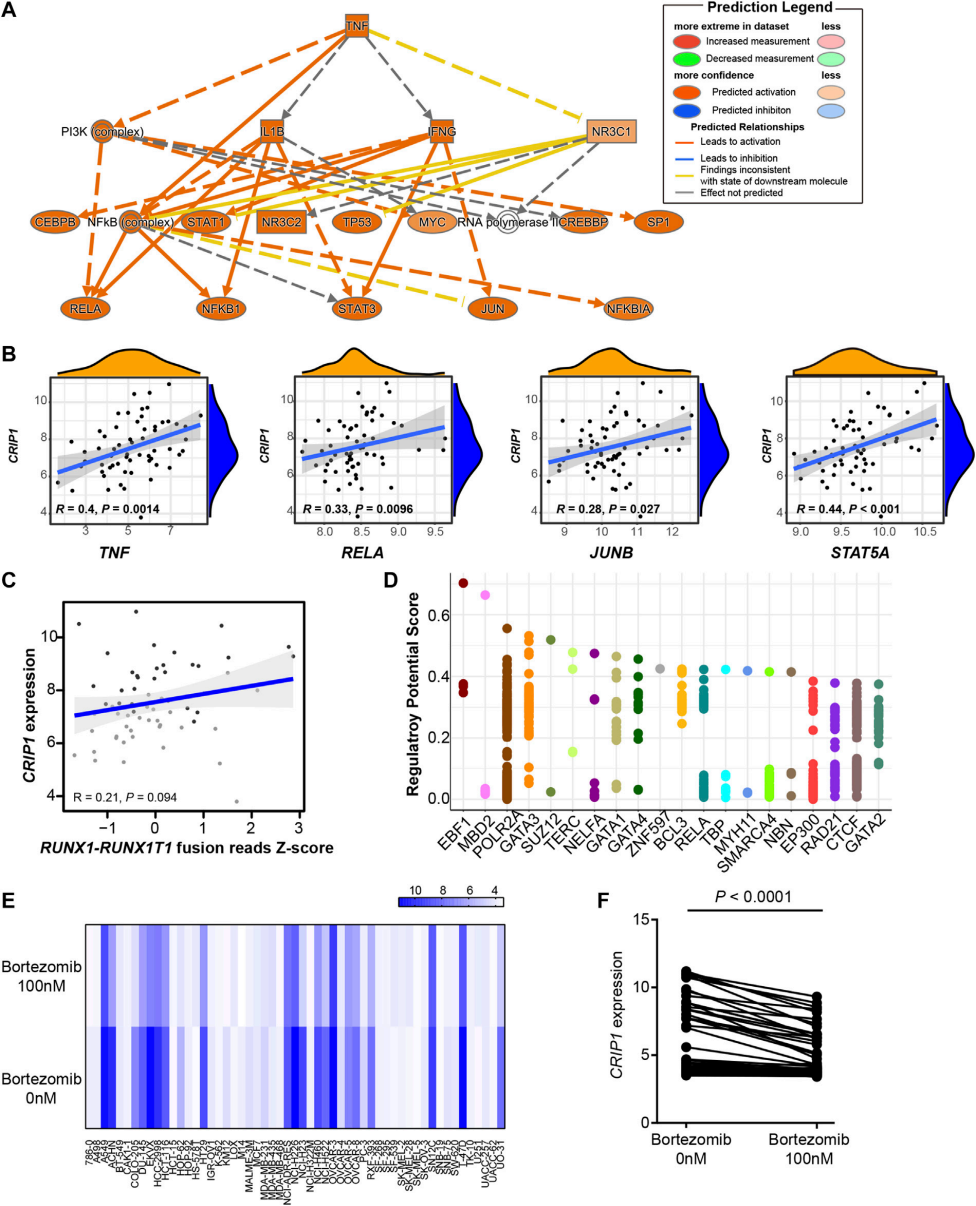
*CRIP1* could be regulated by the tumor necrosis factor (TNF)α–nuclear factor kappa B pathway. **(A)** Ingenuity pathway analysis upstream analysis based on the CD34^+^CD117^dim^ scRNA-seq gene signature from t (8;21) acute myeloid leukemia (AML) patients. **(B)** Correlation analysis of *CRIP1* with *TNF*, *RELA*, *JUNB,* and *STAT5A*. *R* as correlation. Spearman’s correlation analysis and correlation coefficient (*R*) were shown. **(C)** Correlation analysis of *CRIP1* with *RUNX1-RUNX1T1* fusion based on the RNA-seq data of t (8;21) AML patients. Spearman’s correlation analysis and correlation coefficient (*R*) were shown. **(D)** The top 20 putative and potential transcription factors for *CRIP1* from ChIP-seq datasets by Cistrome. The *Y* axis represents the regulatory potential score and the *X* axis represents different factors. Each dot represents a ChIP-seq sample. **(E)** Heatmap of the *CRIP1* expression in different cell lines after exposure to bortezomib (100 nM) or control group (0 nM) for 24 h from GSE116438. Each column represents a cell line. Each row represents the different concentrations of bortezomib, 0 nM (control) or 100 nM. **(F)** Comparison of the *CRIP1* expression in different cell lines after exposure to bortezomib (100 nM) for 24 h from GSE116438. Statistical significance was determined using two-sided Student’s *t*-test.

Next, we explored the regulation network that promoted the high *CRIP1* expression in t (8;21) AML patients. A previous study had compared the gene mutations of RTK/Ras family, transcription factors, epigenetic modifiers, cohesion complexes, or signaling pathways. No significant differences in gene mutations mentioned above were observed between the *CRIP1*-high and -low groups (Li et al., 2021). Considering that the immune pathway was activated, we questioned whether the *CRIP1* was upregulated by the immune regulators of the BM microenvironment. We performed the ingenuity pathway analysis (IPA) using the single-cell RNA-seq (scRNA-seq) data of t (8;21) AML patients, and *TNF* was identified as the main regulator (Figure 2A).

Further correlation analysis demonstrated that the *CRIP1* expression had a significant positive correlation with *TNF* (*R* = 0.4, *p* = 0.0014), *RELA* (*R* = 0.33, *p* = 0.0096), and *JUNB* (*R* = 0.28, *p* = 0.027), all of whom participated in the TNFα–NFκB pathway (Figure 2B). Considering the role of *RUNX1-RUNX1T1* fusion transcript in t (8;21) AML pathogenesis, we questioned whether the *CRIP1* expression was regulated by the fusion gene. As a result, there was no significant correlation with the coefficient of 0.21 with a *p* = 0.094 (Figure 2C), thereby suggesting that the *RUNX1–RUNX1T1* fusion has no direct influence on the *CRIP1* expression.

The putative and potential transcription factors (TF) for the *CRIP1* gene from the ChIP-seq datasets by Cistrome (Mei et al., 2017; Zheng et al., 2019) were then analyzed. Neither TF *RUNX1* (*AML1*) nor *RUNX1T1* (*ETO*) were included as the top putative targets (Figure 2D). Instead, *MBD2*, *RELA*, and *BCL3* were shown as the top putative factors, which belong to the TNFα–NFκB pathway.

To test whether the *CRIP1* expression was regulated by the TNFα–NFκB pathway, we analyzed the drug-induced gene expression change across different cell lines following the exposure to the NFκB inhibitor bortezomib from GSE116438 to mimic the knockdown of the TNFα–NFκB pathway. For the 55 cell lines tested, when treated with bortezomib for 24 h, the *CRIP1* expression was significantly lower (*p* < 0.0001) (Figures 2E,F), which further demonstrated that *CRIP1* was regulated by the TNFα–NFκB pathway.

### Epigenetics Effect on the *CRIP1* Expression in Acute Myeloid Leukemia Patients

**FIGURE 3:**
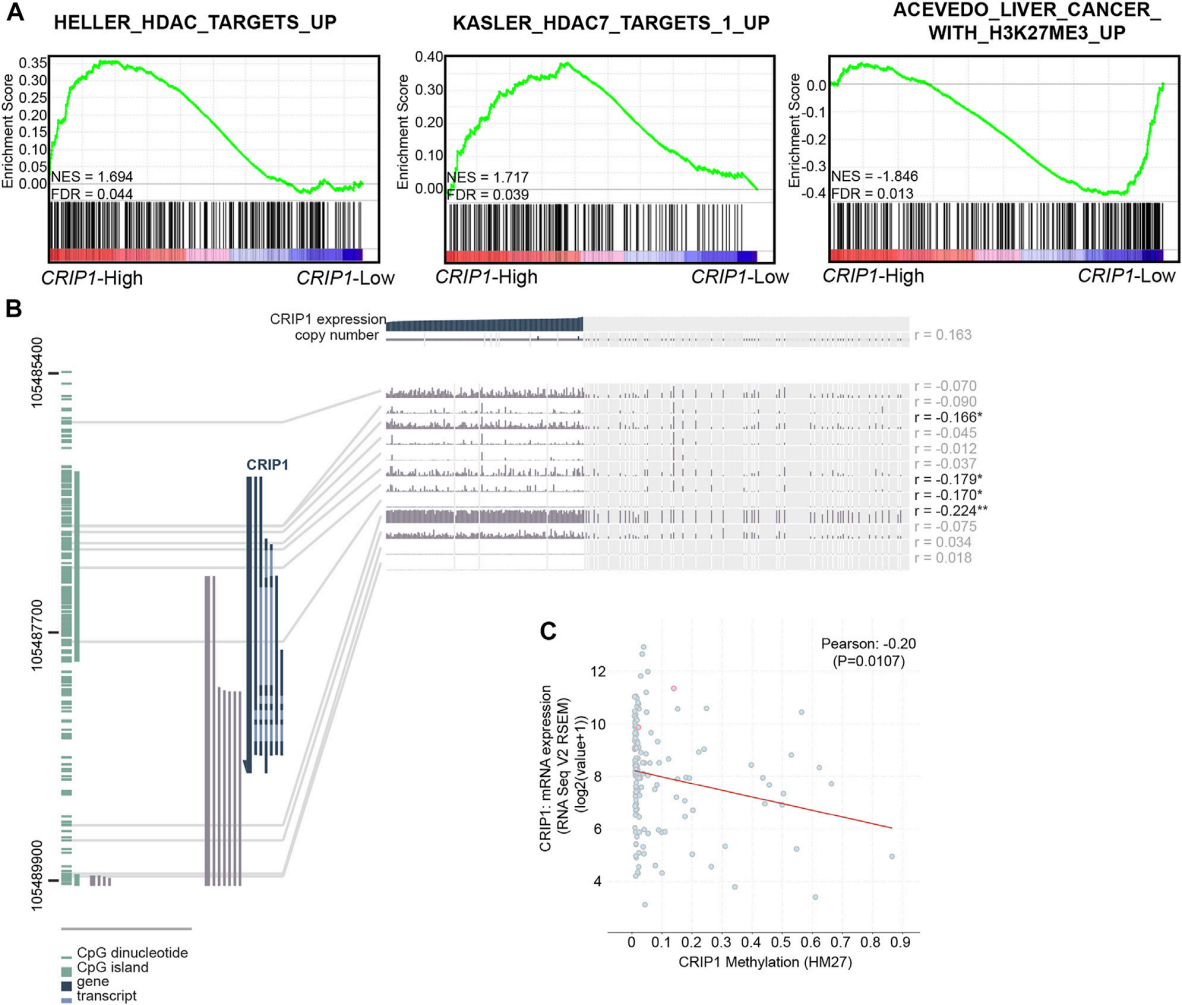
Epigenetic effect on the *CRIP1* expression in acute myeloid leukemia (AML) patients. **(A)** Representative activated HDAC-related pathways in the high- and low-*CRIP1* expressions of t (8;21) AML patients. Normalized enrichment score (NES) and false discovery rate (FDR) values are given. **(B)**. MEXPRESS view of the Cancer Genome Atlas (TCGA) data for *CRIP1* in AML patients. The samples were ordered by *CRIP1* expression. **(C)** Scatter plot of the mRNA expression compared with DNA methylation data (HM27) of *CRIP1* in AML patients with data available (*n* = 159) based on the TCGA database *via* cBioPortal. The correlation of CRIP1 expression with DNA methylation status was shown. Pearson’s correlation analysis and correlation coefficient (*R*) were shown.

We then explored the epigenetics’ influence on the *CRIP1* expression in t (8;21) AML patients. GSEA showed that the *CRIP1*-high group has a significant enrichment in histone deacetylase (HDAC) and HDAC7 targets (Figure 3A). However, the *CRIP1*-low group showed enrichment in H3K27ME3 (Figure 3A).

Next, we explored the relationship between the *CRIP1* expression and the methylation level of the *CRIP1* promoter in AML (Cancer Genome Atlas Research et al., 2013) data using MEXPRESS (Figure 3B). In addition, the correlation of the *CRIP1* expression with the methylation level from the Human Methylation 27k (HM27) platform of TCGA AML patients was analyzed using the cBioPortal (Gao et al., 2013). The DNA methylation level of *CRIP1* was negatively correlated with *CRIP1* expression (Figure 3C). In addition, we identified four CpG probes of *CRIP1* (cg07065217, cg04411625, cg25682097, and 11763800) that were negatively associated with the *CRIP1* gene expression in AML patients.

### *CRIP1* Expression with Immune Regulation was Tested in Other Independent Cohorts

**FIGURE 4:**
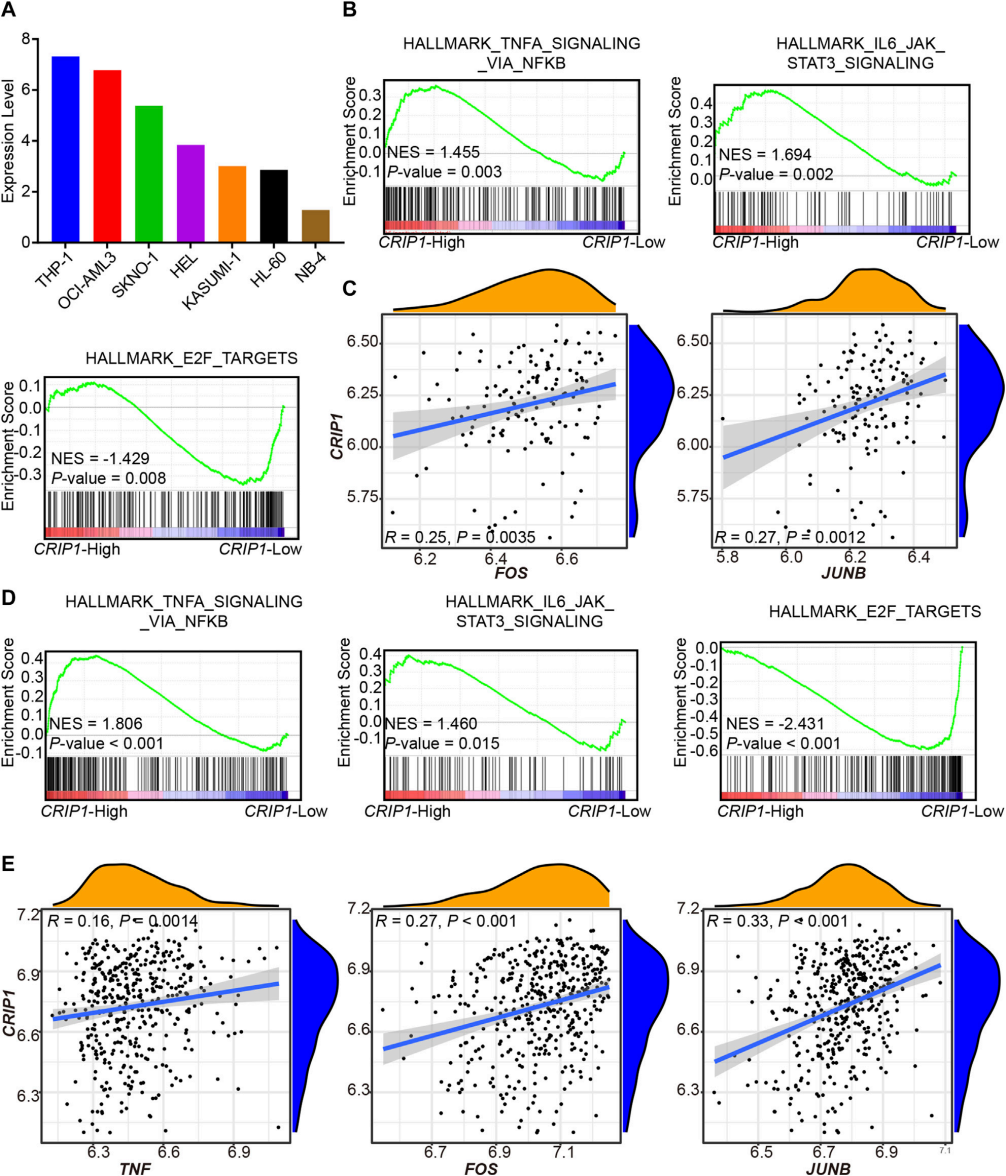
Validation of *CRIP1* expression under the tumor necrosis factor (TNF)α–nuclear factor kappa B (NFκB) pathway in the GSE37642 dataset. **(A)** The CCLE database showed the *CRIP1* expression among the AML cell lines. **(B)** Representative gene set enrichment analysis (GSEA) plots showing the activated immune-related pathways in the high-*CRIP1* expression group from the GSE37642 (GPL 570, *n* = 140) cohort. Normalized enrichment score (NES) and nominal *p* value were given. **(C)** Correlation analysis of the *CRIP1* expression with the transcription factor of the TNFα–NFκB pathway from the GSE37642 (GPL 570, *n* = 140) cohort. Spearman’s correlation analysis and correlation coefficient (*R*) were shown. **(D)** Representative GSEA plots showing the activated immune-related pathways in the high--*CRIP1* expression group from the GSE37642 (GPL 96, *n* = 422) cohort. Normalized enrichment score (NES) and nominal *p* value were given. **(E)** Correlation analysis of the *CRIP1* expression with the transcription factor of the TNFα–NFκB pathway from the GSE37642 (GPL 96, *n* = 422) cohort. Spearman’s correlation analysis and correlation coefficient (*R*) were shown.

**FIGURE 5:**
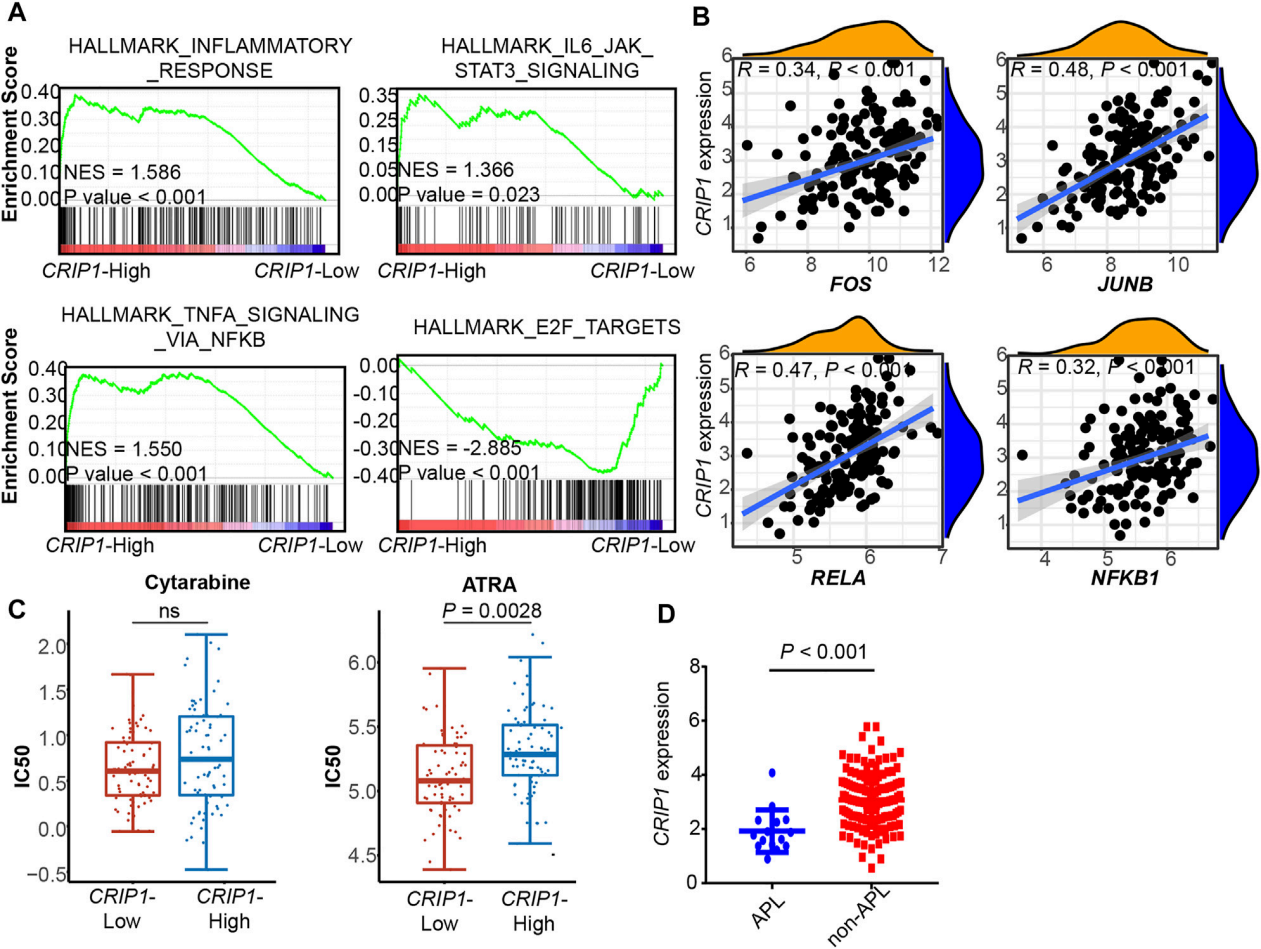
Validation of *CRIP1* expression under the TNFα–NFκB pathway in the TCGA dataset. **(A)** Representative gene set enrichment analysis plots showing the activated immune-related pathways in the high-*CRIP1* expression group from the Cancer Genome Atlas (TCGA) LAML (*n* = 130) cohort. Normalized enrichment score (NES) and nominal *p* value are given. **(B)** Correlation analysis of the *CRIP1* expression with the transcription factor of the TNFα–NFκB pathway from the TCGA LAML (*n* = 130) cohort. Spearman’s correlation analysis and correlation coefficient (*R*) are shown. **(C)** The distribution of IC50 for cytarabine and all-trans retinoic acid (ATRA) between the high- and low-*CRIP1* expression of AML patients. The statistical difference of the two groups was compared using the Wilcox test. **(D)** The comparison of the *CRIP1* expression in APL and non-APL AML patients with data from TCGA LAML. Statistical difference was determined using two-sided Student’s *t*-test.

From the CCLE database, the *CRIP1* expression of AML cell lines was analyzed (Figure 4A) with the highest expression in THP-1 (acute monocytic leukemia) and lowest in NB-4 (acute promyelocytic leukemia, APL). The GSE37642-GPL96, GSE37642-GPL570, and TCGA datasets were used as the validation cohort-1 (Figures 4D,E), validation cohort-2 (Figures 4B,C), and validation cohort-3 (Figures 5A,B).

In the three validation cohorts, we observed the activated enrichment of immune-related pathways in the BM microenvironment, including the TNFA signaling via the NFKB pathway and IL6-JAK-STAT3 signaling pathways in the *CRIP1*-high group. In addition, the *CRIP1*-low group showed a significant enrichment in the E2F target pathway in the three validation cohorts. Further, the immune infiltration cells in these validation cohorts were analyzed using the differential infiltration profile (Supplementary Figures S4, S5). The exhaustion CD8 T cells in t (8;21) AML were not observed in these validation cohorts. This may be due to the complex heterogeneity of AML. Thus, further exploration is required.

In addition, the *CRIP1* expression under the TNFα-NFκB regulation was analyzed. In the three cohorts, the *CRIP1*-high group showed significant enrichment of immune-related pathway. Detailed correlation analysis also demonstrated that the *CRIP1* had a positive correlation with *TNF*, *JUNB,* and *FOS* (Figures 4C,E, 5B).

### *CRIP1* Expression was Higher in Non-Acute Promyelocytic Leukemia Patients than Acute Promyelocytic Leukemia Patients

The IC50 distribution of cytarabine and all-trans retinoic acid (ATRA) was analyzed using data from the GDSC dataset. The *CRIP1*-high group had a trend of higher IC50 of cytarabine (Figure 5C), though it did not reach statistical significance. In addition, the *CRIP1*-high group had a significantly higher IC50 of ATRA (*p* = 0.0028).

In addition, we compared the *CRIP1* expression in APL and non-APL AML patients using the TCGA AML data. Compared with APL patients, non-APL AML patients had a significantly higher *CRIP1* expression (*p* < 0.001) (Figure 5D **)**.

The higher *CRIP1* expression in non-APL patients made us explore the pattern of *CRIP1* expression in normal myeloid differentiation. Using data from BloodSpot (Bagger et al., 2019), a database of healthy and malignant hematopoiesis, late promyelocyte did have a rather low *CRIP1* expression (Supplementary Figures S6A,B). In addition, we observed a higher *CRIP1* expression in mature monocytes compared to the hematopoietic stem and progenitor cells, including multipotential progenitors, common myeloid progenitor cell, and granulocyte monocyte progenitors (Supplementary Figure S6A).

## Discussion

This study examined the genetic and epigenetic regulation of *CRIP1* expression in AML patients via multidimensional analyses of gene expression and methylation data. It further explored the immune regulation of *CRIP1* expression, especially the TNFα–NFκB signaling pathway in AML. Our preliminary research might provide new insights for exploring improvements in AML treatment.

The role of *CRIP1* expression in AML patients has been rarely reported and is largely unknown. In fact, *CRIP1* was reported to have tumor-specific oncogenic or tumor-suppressive properties (Sun et al., 2021). In colorectal cancer, *CRIP1* could facilitate the 5-FU drug resistance by downregulating the Fas and Fas-mediated apoptosis-related proteins’ expression (Zhang et al., 2019). However, in breast cancer patients, low *CRIP1* expression could enhance cell proliferation and invasion by enhancing the mitogen-activated protein kinase phosphorylation and reducing the CDC2 phosphorylation (Ludyga et al., 2013). In a previous study, we demonstrated that *CRIP1* was highly expressed in AML patients, including the M0–M7 subtype (Li et al., 2021). The prognostic value of *CRIP1* expression in t (8;21) AML was first reported by our group (Li et al., 2021). In this work, based on the scRNA-seq and gene expression data of t (8;21) AML patients, we reported that the *CRIP1* was regulated by the TNFα–NFκB pathway. In addition, the *CRIP1*-low group demonstrated significant enrichment of E2F target pathway in the t (8;21) AML cohort (OEP000629) and three other validation cohorts. This activation of E2F targets was consistent with the enrichment result of the CD34^+^CD117^bri^ population, which showed a lower *CRIP1* expression in our previous studies (Jiang et al., 2020).

Previous studies have shown that AML leukemia cells can produce endogenous TNFα, which then activated the downstream NFκB-signaling pathway, thereby resulting in leukemia cell proliferation and drug resistance (Hemmati et al., 2017). The transcription factor *AML1* inhibits the NFκB-signaling pathway by interacting with the IκB kinase complex, while the fusion gene *AML1-ETO* lacks the inhibitory effect on the NFκB-signaling pathway (Lin et al., 2017). In this work, combined with ChIP-seq data, the *CRIP1* expression was abnormally activated by the TNFα–NFκB signaling pathway, which promoted the proliferation of leukemia cells, thereby resulting in relapse and drug resistance of t (8;21) AML patients. This was further validated in two independent cohorts of AML patients.

Through further exploration of the expression pattern during myeloid differentiation, we found that at the late promyelocyte stage, the *CRIP1* expression was lower, while the monocyte had a rather high level of *CRIP1* expression. However, the role of *CRIP1* in the myeloid differentiation still needs further exploration.

Up to date, the treatment of AML still faces challenges of drug resistance and relapse. Multiple studies have shown that immunotherapy is playing an integral role in AML (Berger et al., 2008; Zhang et al., 2009; Kasakovski et al., 2018). The single-arm, phase 2 study explored the efficiency of adding PD-1 monoclonal antibody (nivolumab) to the standard IA regimen for AML (idarubicin combined with cytarabine). It was reported to reduce the recurrence rate and improve the prognosis of AML (Ravandi et al., 2019). However, there are still patients with a poor response to immunotherapy. Through the immune infiltration of t (8;21) AML patients, we found that the *CRIP1*-high group had a higher proportion of exhausted CD8 T cells. In fact, the dysfunction of immune cells, especially T cells, was revealed to affect the prognosis of patients with tumors, including nonsmall cell lung cancer, colorectal cancer, and melanoma (Guo et al., 2018; Li et al., 2019; Zhang et al., 2020). However, the immune infiltration analysis of gene expression profile from other validation cohorts did not observe the same pattern of higher exhausted CD8 T cells in AML patients. An in-depth study of the immune regulation mechanism of AML will help improve its efficacy.

In conclusion, our data provide a comprehensive overview of the regulation of *CRIP1* expression in AML patients. The evaluation of the TNFα-NFκB signaling pathway as well as the immune heterogeneity might provide new insights for exploring improvements in AML treatment.

## Data Availability

The original contributions presented in the study are included in the article/supplementary material, further inquiries can be directed to the corresponding authors.
