# Supplementary figures and images for: Comprehensive Analysis of CRIP1 Expression in Acute Myeloid Leukemia
Source: Front Genet. 2022 Jul 22;13:923568. doi: 10.3389/fgene.2022.923568 (PMC9354089; doi:10.3389/fgene.2022.923568)

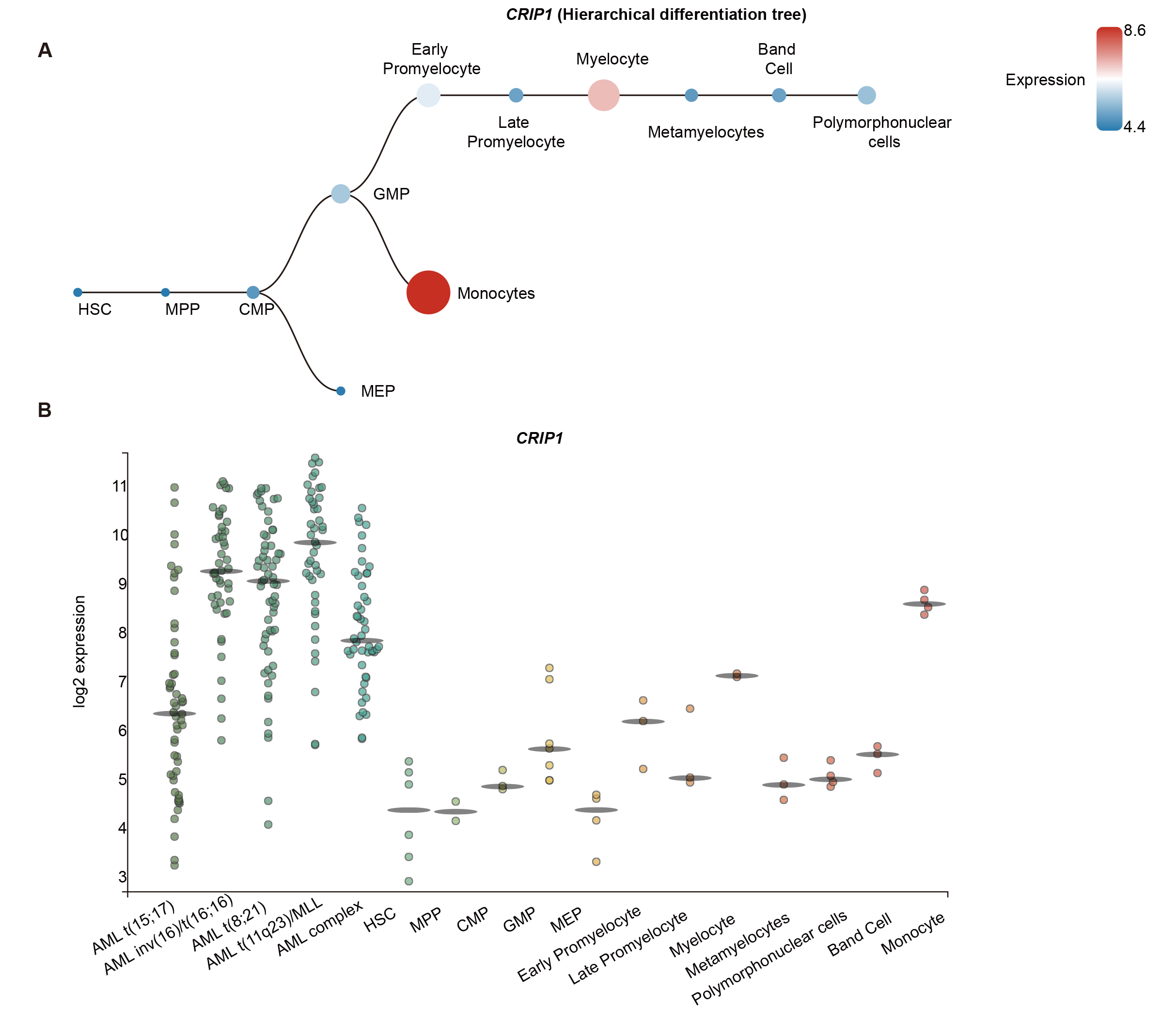

Supplement: Supplementary file 1 [file Image6.TIF]

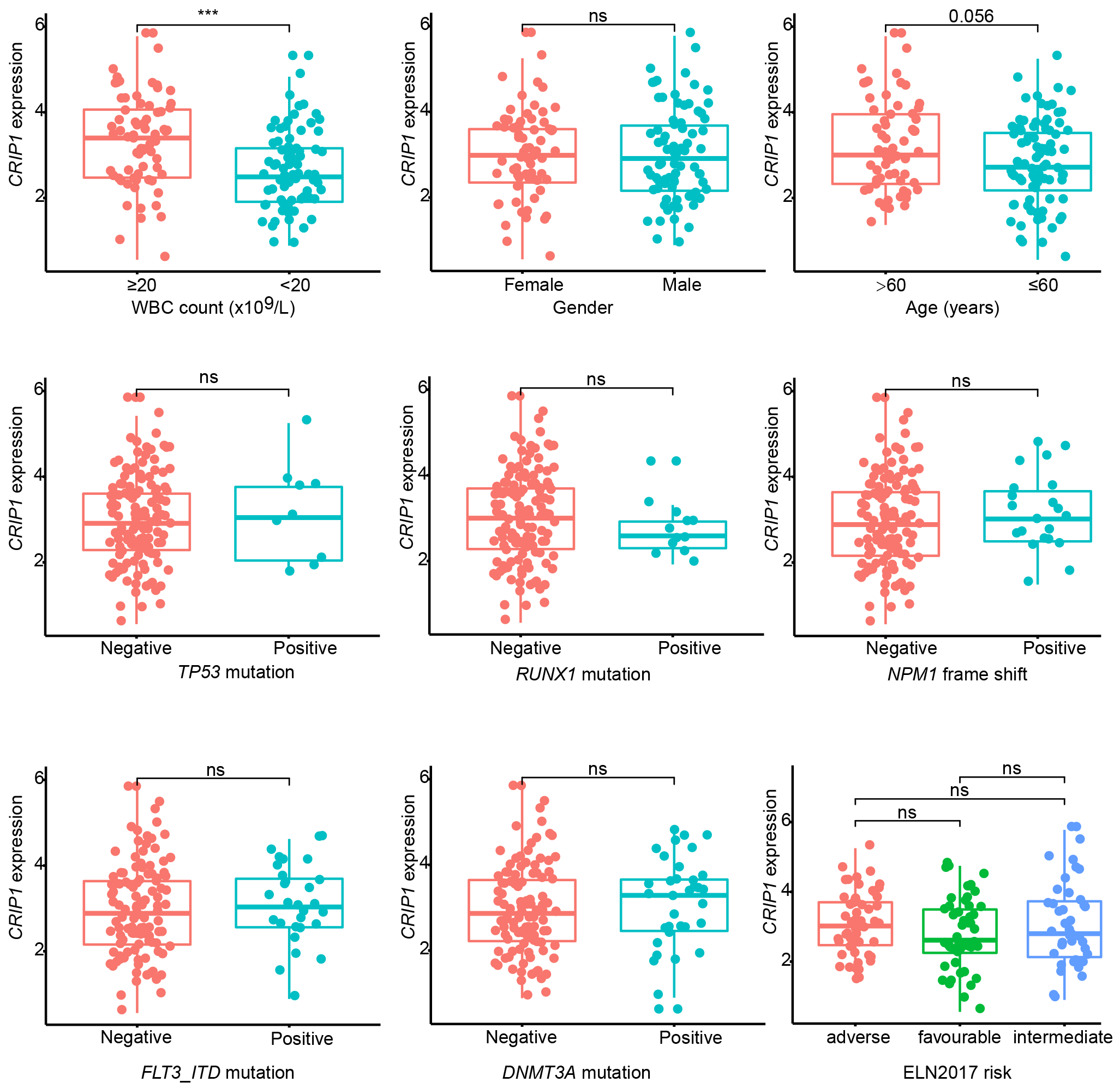

Supplement: Supplementary file 2 [file Image3.TIF]

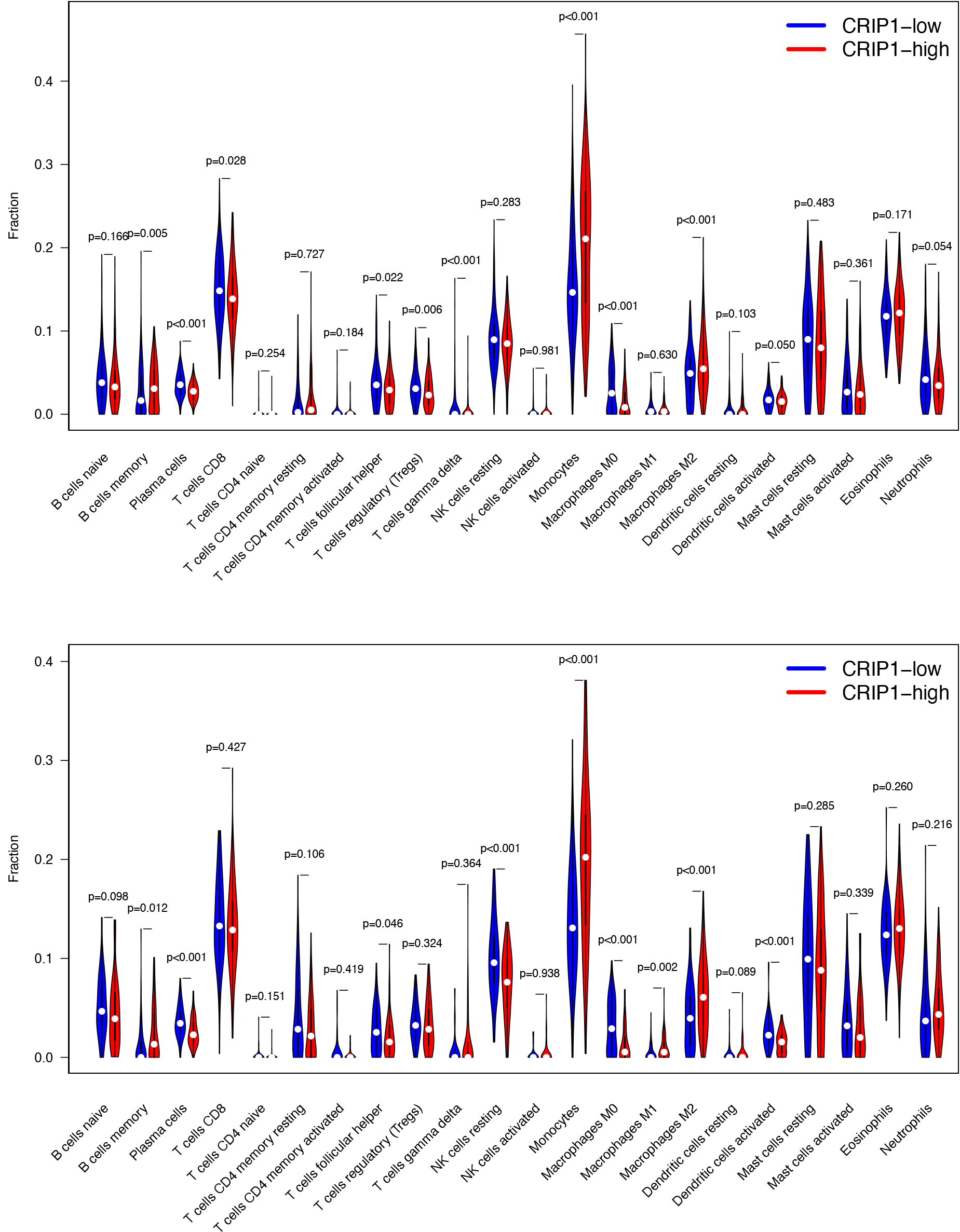

Supplement: Supplementary file 3 [file Image4.TIF]

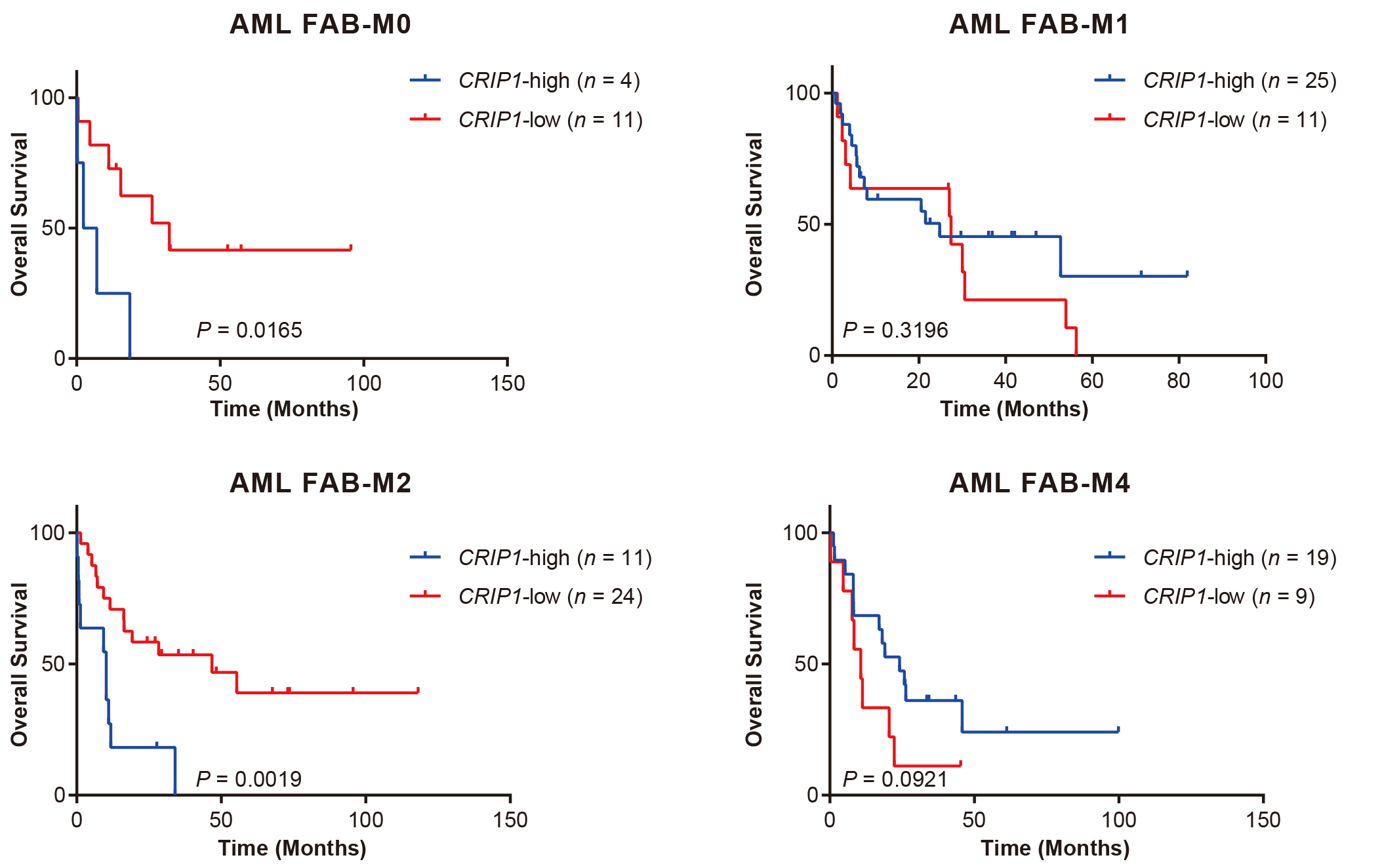

Supplement: Supplementary file 4 [file Image2.TIF]

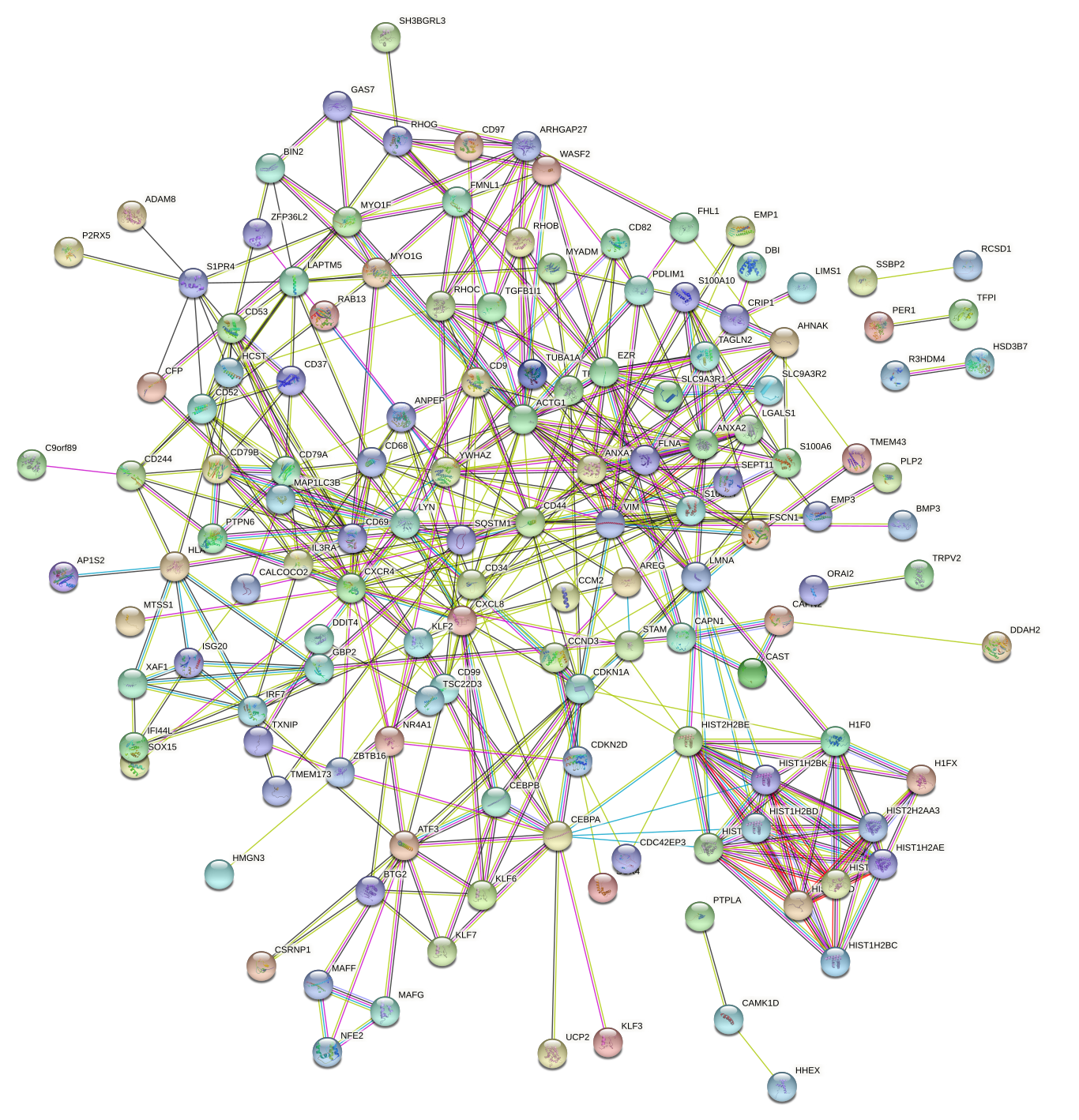

Supplement: Supplementary file 5 [file Image1.TIF]

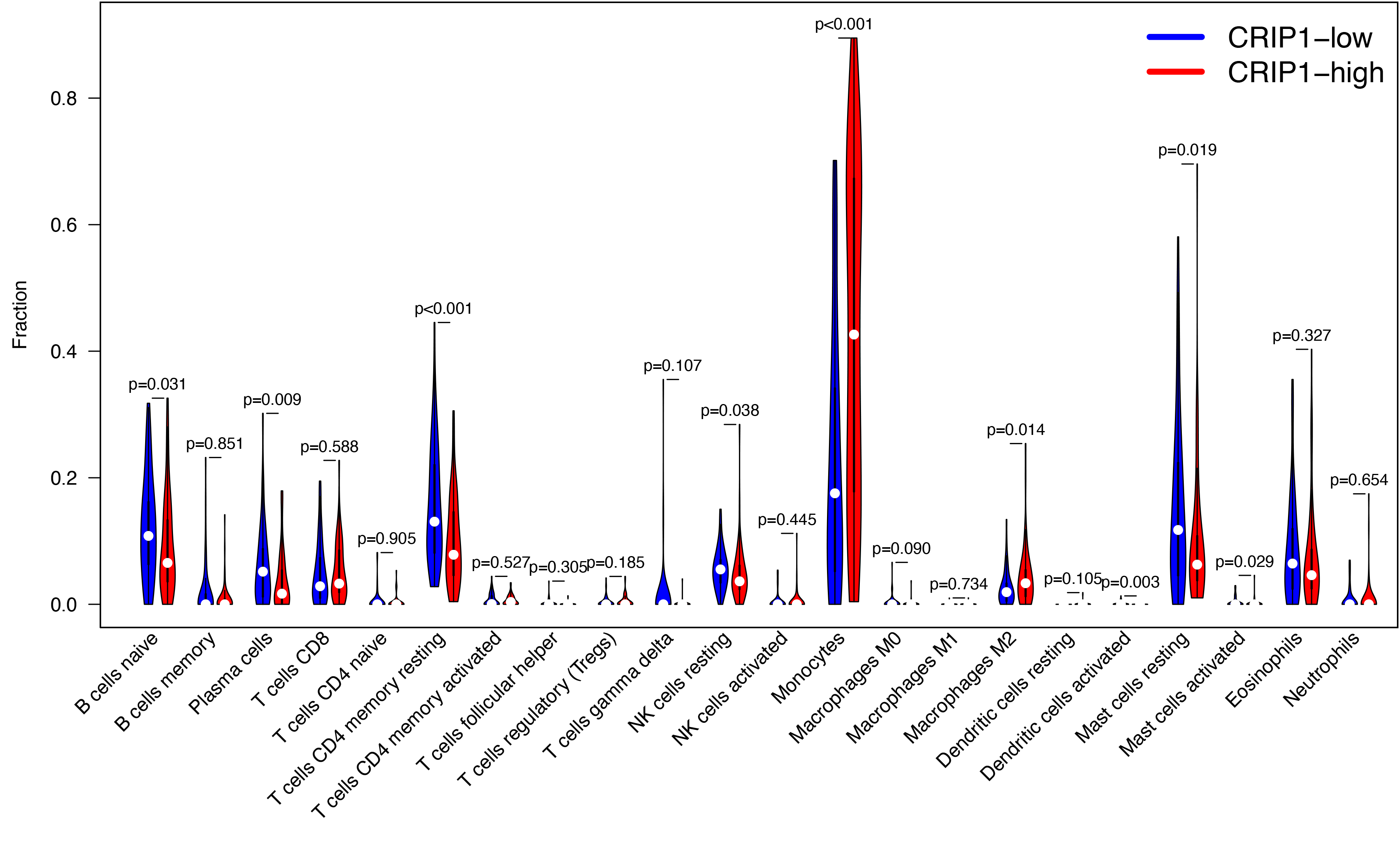

Supplement: Supplementary file 8 [file Image5.TIF]
