# Supplementary material for: Comprehensive Analysis of CRIP1 Expression in Acute Myeloid Leukemia
Source: Front Genet. 2022 Jul 22;13:923568. doi: 10.3389/fgene.2022.923568 (PMC9354089; doi:10.3389/fgene.2022.923568)
Supplement: Supplementary file 6 [file DataSheet1.pdf]

Supplementary File

Figure S1. PPI construction based on the CD34$^+$CD117^{dim} gene signature.

Minimum required interaction score of 0.400 with disconnected nodes in the network hidden. Line colors indicated the type of interaction evidence.

Figure S2. Comparison of overall survival between patients with high- and low- *CRIP1* expression in each FAB subtype among the TCGA AML cohort.

Survival curves were estimated using the Kaplan-Meier method and compared using the log-rank test.

Figure S3. Comparison of *CRIP1* expression in each subgroup classified by the clinical characteristics or genetic mutations among the TCGA AML cohort.

*, $P < 0.05$; **, $P < 0.01$; ***, $P < 0.001$. ns, not significant. Statistical significance was determined by two-sided Wilcoxon test.

Figure S4. Immune infiltration analysis of GSE37642 datasets based on the CIBERSORT algorithm.

(Upper Panel). Proportion of immune infiltrated cells between high- and low- *CRIP1* expression of GSE37642 (GPL 96, n = 422) cohort based on the CIBERSORT algorithm. *, $P < 0.05$; **, $P < 0.01$; ***, $P < 0.001$. Statistical significance was determined by two-sided Wilcoxon test.

(Lower Panel). Proportion of immune infiltrated cells between high- and low- *CRIP1* expression of GSE37642 (GPL 570, n=140) cohort based on the CIBERSORT algorithm. *, $P < 0.05$; **, $P < 0.01$; ***, $P < 0.001$. Statistical significance was determined by two-sided Wilcoxon test.

Figure S5. Immune infiltration analysis of the TCGA AML datasets based on the CIBERSORT algorithm. *, $P < 0.05$; **, $P < 0.01$; ***, $P < 0.001$. Statistical significance was determined by two-sided Wilcoxon test.

Figure S6. Comparison of *CRIP1* expression during myeloid differentiation based on the BloodSpot database.

A. Hierarchical differentiation tree of *CRIP1* expression during myeloid differentiation.

B. Comparison of *CRIP1* expression in each stage of myeloid differentiation.

HSC (Hematopoietic stem cell), MPP (Multipotential progenitors), CMP (Common myeloid progenitor cell), GMP (Granulocyte monocyte progenitors) and MEP (Megakaryocyte-erythroid progenitor cell).

Table S1. The signature of CD34$^+$CD117^{dim} populations extracted from previous scRNA-seq data.
